# ACER3-related leukoencephalopathy: expanding the clinical and imaging findings spectrum due to novel variants

**DOI:** 10.1186/s40246-021-00345-0

**Published:** 2021-07-19

**Authors:** Ali Zare Dehnavi, Erfan Heidari, Maryam Rasulinezhad, Morteza Heidari, Mahmoud Reza Ashrafi, Mohammad Mahdi Hosseini, Fatemeh Sadeghzadeh, Mohammad-Sadegh Fallah, Noushin Rostampour, Amir Bahraini, Masoud Garshasbi, Ali Reza Tavasoli

**Affiliations:** 1 Myelin Disorders Clinic, Pediatric Neurology Division, Children’s Medical Center, Pediatrics Center of Excellence, Tehran University of Medical Sciences, Tehran, Iran; 2 Department of Medical Genetics, Faculty of Medical Sciences, Tarbiat Modares University, Tehran, Iran; 3 Kawsar Human Genetics research center, Tehran, Iran; 4 Metabolic Liver Disease Research Center, Isfahan University of Medical Sciences, Isfahan, Iran; 5 Department of Human Genetics, Graduate School of Public Health, University of Pittsburgh, Pittsburgh, PA USA; 6 KaryoGen, Isfahan, Iran

**Keywords:** Leukodystrophy, Alkaline Ceramidase 3, Whole-exome sequencing, Magnetic resonance imaging

## Abstract

**Background:**

Leukodystrophies are the main subgroup of inherited CNS white matter disorders which cause significant mortality and morbidity in early years of life. Diagnosis is mostly based on clinical context and neuroimaging findings; however, genetic tools, particularly whole-exome sequencing (WES), have led to comprehending the causative gene and molecular events contributing to these disorders. Mutation in Alkaline Ceramidase 3 (*ACER3*) gene which encodes alkaline ceramidase enzyme that plays a crucial role in cellular growth and viability has been stated as an uncommon reason for inherited leukoencephalopathies. Merely only two *ACER3* mutations in cases of progressive leukodystrophies have been reported thus far.

**Results:**

In the current study, we have identified three novel variants in ACER3 gene in cases with new neurological manifestations including developmental regression, dystonia, and spasticity. The detected variants were segregated into family members.

**Conclusion:**

Our study expands the clinical, neuroimaging, electroencephalographic, and genetic spectrum of *ACER3* mutations. Furthermore, we reviewed and compared the findings of all the previously reported cases and the cases identified here in order to facilitate their diagnosis and management.

**Supplementary Information:**

The online version contains supplementary material available at 10.1186/s40246-021-00345-0.

## Background

Leukodystrophies are a heterogeneous group of neurogenetic disorders which affect either myelin production or maintenance of the myelin mainly in the central nervous system (CNS). They are associated with substantial morbidity and mortality in children [1, 2]. The incidence of leukodystrophies has been estimated to be 1 in 7663 live births or 3.01/1000,000 in children under the age of 18 years depending on the applied epidemiological methods [3, 4].

The diagnostic approach is usually based on the precise review of neurologic and non-neurologic symptoms and recognition of white matter involvement patterns in brain magnetic resonance imaging (MRI) [5]. Molecular studies especially whole-exome sequencing analysis are essential diagnostic tools to identify the causative genes [6]. Such studies in some cases may result in the expanding of the clinical and imaging findings in patients and a wider perception of cellular aspects of the known genes.

*ACER3* is responsible for coding the alkaline ceramidase enzyme which is responsible for the conversion of ceramide to sphingosine. Ceramide is a critical building block for sphingolipids that are required for both growth and viability of cells. On the other hand, Ceramide plays as a sensor of stress that controls several cellular reactions such as growth arrest or cell death. For cellular well-being, cellular levels of sphingosine, which is the product of normal ceramidase activity, and sphingosine phosphate are important [7, 8]. Thus, human alkaline ceramidase plays an important role in cell response to stress by controlling the hydrolysis process of ceramides in cells [9].

Mutated *ACER3* which results in the accumulation of multiple sphingolipids and long-chain ceramides in brain and blood circulation has been reported to be associated with abnormal brain white matter signals. This phenomenon potentially accounts for a rare form of *ACER3*-related genetic leukoencephalopathy [10, 11]. Faint abnormal white matter signals in deep and periventricular areas, thinning of corpus callosum, mild supratentorial and cerebellar atrophy, and ventriculomegaly are the imaging findings that have been reported in association with mutations in *ACER3* coding alkaline ceramidase (Table 1).

**Table 1:** Clinical features of patients harboring the *ACER3* gene variants

Variables	Present study P1	Present study P2	Present study P3	Edvardson study*	Edvardson study*	Huseyin Kilic study**
**Sex**	Female	Male	Male	Female	Male	Male
**Consanguinity**	First cousin	First cousin	First cousin	Remotely consanguineous	Remotely consanguineous	First cousin
**Head circumference (HC)**	Normal HC at first but late-infantile-onset microcephaly	Normal HC	Normal HC at first but late-infantile-onset microcephaly	Late-onset macrocephaly	Late-onset macrocephaly	Normal (at 1 month), microcephaly (at 3 years old)
**Pregnancy history**	None	None	Preeclampsia	None	None	Not mentioned
**Family history of additional affected sibling**	−	−	−	+	+	−
**Age at disease onset (M)**	5–6 months	11 months	6 months	6–13 months	6–13 months	12 months
**Condition/age at the end of the study (Y)**	Died at 3 years old	3 years old	1.66 years	13 years old	11 years old	3 years old
**The best speech ability**	2–3 words	5–10 words	Less than 10 incomplete words	5–10 words	5–10 words	3–5 words
**Neurologic examination**	Generalized dystonia, limb spasticity, hyperreflexia, truncal hypotonia, upward plantar reflex, opisthotonus	Distal dystonia, limb spasticity, hyperreflexia, upward plantar reflex, tripod sitting position, clonus	Dystonia, limb spasticity, hyperreflexia, upward plantar reflex	Dystonia, appendicular spasticity, areflexia, truncal hypotonia	Dystonia, appendicular spasticity, areflexia, truncal hypotonia	Dystonia, spasticity without areflexia, truncal hypotonia, ankle clonus
**Systemic examination**	Low set ears, high arched palate, beaked nose, flat nasal bridge, thick eyebrows	Normal	Normal	Low set ear, thick eyebrows, sloping forehead, prominent nose, short stature, flat philtrum, prominent lower lip	Low set ear, thick eyebrows, sloping forehead, prominent nose, short stature, flat philtrum, prominent lower lip	Coarse facial appearance, mild hirsutism, low anterior hair line prominent forehead, elongated face, bilateral epicanthal folds long eyelashes, low set ears, short philtrum prominent lower lip
**Feeding condition**	Feeding with gastrostomy tube (PEG)	No problem in feeding	No problem in feeding	Feeding with gastrostomy tube (PEG)	Feeding with gastrostomy tube (PEG)	Not mentioned
**Respiratory support**	Mechanical ventilation	No problem in breathing	No problem in breathing	Mechanical ventilation	Mechanical ventilation	Not mentioned
**Basic metabolic tests**	All NL except for high lactate	All NL	All NL	All NL except for high lactate	Not available	Not available
**EEG findings**	Low-voltage background, generalized slowing, no epileptic discharge activity	Normal	Not available	Generalized slowing without epileptic activity	Not available	Not available
**EMG-NCV findings**	Not available	Not available	Generalized sensory polyneuropathy	Not available	Not available	Sensory neuropathy without any motor neuron conduction abnormality
**Brain MRI**	MRI at age 18 months: Mild supratentorial atrophy, thinning of corpus callous, abnormal signal of posterior periventricular white matter, deep Sylvian fissure, ventriculomegaly Second brain MRI at age 27 months: the same findings with progressive atrophy	MRI at age 18 months: Increased signal intensity in white matter of the trigone area	MRI at 1 year old: normal MRI at age 18 months: Delayed myelination With posterior deep and periventricular white matter abnormalities	At 1 and 2 years: normal At 7 years: diffuse supratentorial and infratentorial atrophy, thinning of corpus, abnormal white matter signal changes	Not available	At 7 months old: normal At age 22 months: increased white matter signals on periventricular and parietal deep white matter At age 32 months: widespread increase in white matter intensity, ventricular enlargement, thinning of corpus callosum, cerebral and cerebellar atrophy
**Variant**	c.53T>C	c.292>C	c.566G>A	c.98A>G	c.98A>G	c.233G>A
**Amino acid change**	p.leu18pro	p.Tyr98His	p.Trp189X	p.Glu33Gly	p.Glu33Gly	p.Trp78X

So far, only two mutations in *ACER3* have been reported. Herein, we present three additional variants as a cause for ACER3-related leukoencephalopathy in 3 cases and discuss their clinical course and serial imaging findings in detail which has resulted in expanding the spectrum of this disorder.

## Results

### Clinical assessments

#### Case 1

The patient was a 3-year-old female who presented to Myelin Disorders Clinic due to progressive neurologic regression which was first noticed at the age of 18 months. She was the only child of a consanguineous parent which was born at 39 weeks of gestational age through a Cesarean section following an uneventful delivery with birth weight and head circumference of 3550 g (*Z*-score +1SD) and 35.5 cm (*Z*-score +1SD), respectively. Her mother had received good prenatal care without any significant problems. The patient achieved her developmental milestones normally until the age of 5–6 months. At this age, she had acceptable social interactions and was able to hold her head up, could sit with one hand support, could say mama and dada, and was able to recognize her parents. Since then, developmental arrest and neurologic regression began firstly by feeding and swallowing problems, four limb dystonia, severe para-spinal muscle spasm, and dystonia causing severe opisthotonus followed by scissoring position and spasticity in lower limbs. At the age of 1.5 years, she needed to be supported significantly in order to sit. The maximum speech ability was not exceeding 2–3 incomplete words’ expression, and she had several problems with feeding and swallowing as she was fed with mixed ground foods and clear drinks. She had undergone several occupational therapy sessions which were ordered by her primary care physician. She faced remarkable and more rapid neurologic regression after routine vaccination at the age of 18 months, which led to considerable feeding difficulties, the inability of word expression, and poor head and neck control. Then, she was referred to our clinic. On examination, she had low set ears, high arch palate and beaked nose, flat nasal bridge, and thick eyebrows in addition to significant four limb dystonia, truncal hypotonia, and a noticeable degree of spasticity in her distal parts of both upper and lower limbs.

Upon the follow-up visit at 27 months of age, her main clinical findings were four limb spasticity, severe generalized dystonia, hyperreflexia with + 3 deep tendon reflexes (DTRs), and upward plantar reflex. Her weight was 8 kg (less than 3rd centile, *Z*-score −3SD) and HC was 47 cm (*Z*-score 0SD). She was taking the following medications: diazepam, tizanidine, baclofen, melatonin, and trihexyphenidyl.

**Fig. 1:**
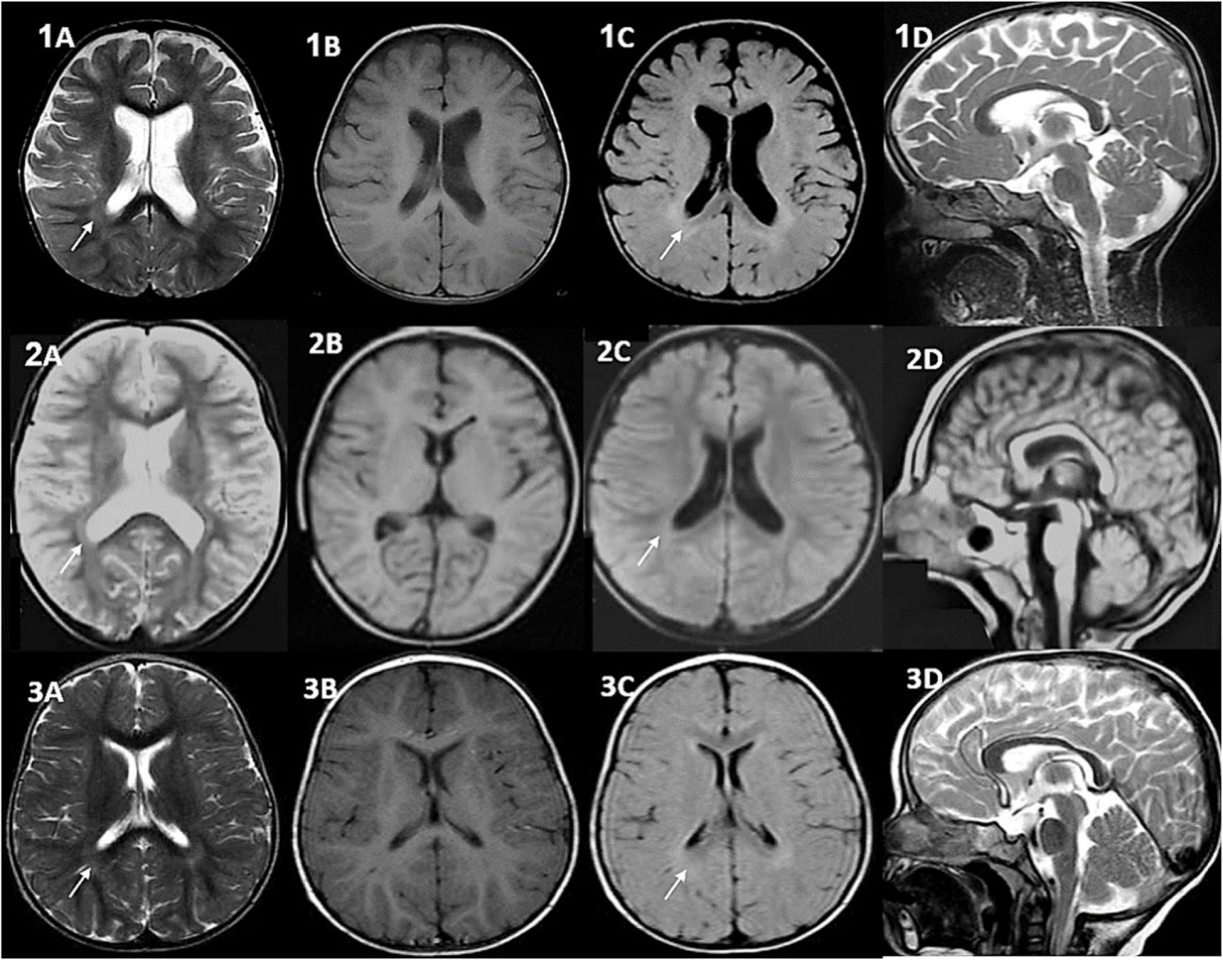
Brain MRI sequences of P1 (1A–D), P2 (2A–D), and P3 (3A–D) without Gad at the age of 18 months. Posterior periventricular and deep white matter signal changes in axial T2-weighted and FLAIR images (1A, 1C–2A, 2C–3A, 3C white arrows), deepening of the Sylvain fissures (1A), mild supratentorial atrophy and exvacue ventriculomegaly (IA, 1C, 2A), near-normal appearance of axial and sagittal T1-weighted imaged (1B–2B–3B, 2D), and thinning of the corpus callosum (1D, 3D) were the most prominent imaging findings. In addition, delayed myelination in subcortical white matter is seen at the age of 18 months (3A)

Basic metabolic tests indicated a high plasma lactate level of 39.7 mmol/L (NL < 16 mmol/L), while the results of serum ammonia, homocysteine, and metabolic screen (MS/MS) were all normal. The first brain magnetic resonance imaging (MRI) which was done at the first visit showed abnormal deep white matter signal changes especially in the posterior periventricular area of T2-weighted and FLAIR sequences, deepening of the Sylvain fissures, and mild supratentorial atrophy, in addition to exvacue ventriculomegaly (Fig. 1 (1A, 1C white arrows)), near-normal appearance of T1-weighted sequence (Fig. 1 (1B)), and thinning of the corpus callosum (Fig. 1 (1D)).

Imaging findings in the second brain MRI at the age of 27 months were similar to the first MRI but the severity of atrophy had increased in both the supratentorial area and cerebellar vermis (not shown).

According to the progressive patient’s clinical scenario, normal basic metabolic studies, and parents’ consanguinity, whole-exome sequencing (WES) was considered to diagnose a possible underlying neurodegenerative disease. In the follow-up visit, at the age of 29 months, her parents reported infrequent focal seizures; therefore, levetiracetam was prescribed for her to control the seizures. The patient was admitted into the hospital with clinical manifestations of aspiration pneumonia at the ages of 32 and 35 months due to a progressive worsening of her feeding condition. During the second hospitalization, her spasms and dystonia were aggravated which led to severe respiratory distress and admission to the pediatric intensive care unit (PICU) and tracheal intubation. The laboratory tests showed a high serum lactate level of 39 mmol/L (NL < 16 mmol/L), again, while serum ammonia, homocysteine, arterial blood gas (ABG), and cerebrospinal fluid (CSF) analysis results were normal. Electroencephalogram (EEG) revealed abnormal findings due to the appearance of generalized slowing and low-voltage background without any paroxysmal epileptiform discharge. These findings were compatible with a diffuse encephalopathic process pattern. Percutaneous endoscopic gastrostomy (PEG) was placed for the patient to facilitate her difficulty feeding within a short time after discharge.

Upon the last visit, at age of 36 months, her problems especially limb spasticity and dystonia were still present but the feeding problem had been ameliorated though she had gained a weight of 300 g over 1 month after discharge. Her seizures were controlled by oral levetiracetam and rectal diazepam as needed. Her weight was 9 kg (*Z*-score −3SD) and her head circumference was 48 cm (*Z*-score 0SD). Unfortunately, she died 1 month later due to cardiorespiratory arrest at home.

#### Case 2

A 3-year-old male was referred to Myelin Disorders Clinic who exhibited developmental motor and speech regression which was started at 11 months of age without any precedent event.

He was born at 36 weeks of gestation after a trouble-free vaginal delivery from a first cousin’s parent. His birth weight and head circumference (HC) were 3700 g (*Z*-score + 1) and 33 cm (*Z*-score 0), respectively. The patient achieved neck holding at the age of 3 months, independent sitting at the age of 8 months, walking with support at the age of 11 months, and a few word expression and good social contact at the age of 12 months.

His parents had first noted limb jerking movements during sleep at the age of 11 months that was treated by clonazepam. Then after, he experienced progressive lower limb spasticity and fine and gross motor regression succeeded by speech regression but cognition ability was partially spared.

On physical examination, he was alert with a HC of 49.5 cm (*Z*-score 0) and a weight of 14 kg (*Z*-score 0). He had an acceptable visual fix and follow, normal horizontal and vertical eye movements, and normal pupils’ reaction to light and normal gag reflex. He had no dysmorphic feature. Cranial nerve examination was normal. Deep tendon reflexes of upper extremities were 3+ and lower extremities were 4+ with clonus. Plantar reflexes were bilaterally upward. Distal four limb dystonia was also seen. He had a tripod sitting position. His gross motor ability was estimated 3 out of 5 based on the Gross Motor Function Classification System (GMFCS) scale. The skin integrity was normal without any congenital or acquired rash. No organomegaly was detected.

His older brother had a similar clinical scenario which was started after a normal growth and developmental course until late infancy, but after that, his motor and then speech milestones were regressed. Infrequent non-febrile seizures were started at the age of 3 years old. He eventually died after a deteriorative clinical course at 19 years of age with clinical presentations of severe appendicular spasticity, generalized dystonia, feeding problems, and respiratory failure. He had not been diagnosed with any specific neurologic disorder in spite of extensive metabolic studies.

In proband case, basic laboratory and metabolic results including thyroid and liver function tests, serum ammonia and lactate, urine organic acid profiles, metabolic screen (MS/MS), and serum acylcarnitine profile were all normal. Electroencephalogram (EEG) and karyotype were both normal. Clonazepam, tizanidine, biotin, CoQ10, and Omega-3 syrup were prescribed for him and regular occupation therapy was started.

His brain MRI at the age of 18 months showed a faint increased signal intensity of white matter of the posterior periventricular area (Fig. 1 (2A, C white arrows)) and no significant finding in axial and sagittal T1-weighted images (Fig. 1 (2B,D)).

#### Case 3

The patient was a 20-month-old male who was referred to Myelin Disorders Clinic due to motor milestones delay and neurologic regression which was first noted at 6 months of age by his parents. He was born at term at 39 weeks of gestation through normal vaginal delivery from consanguineous parents. His mother had taken good prenatal care; however, she experienced preeclampsia during her final pregnancy trimester. The birth weight and head circumference (HC) of the patient were 3350 g (*Z*-score 0SD) and 34 cm (*Z*-score 0SD), respectively. Shortly after birth, he was admitted to the neonatal intensive care unit (NICU) due to a low APGAR score at 5 min of age, not crying, and possible birth asphyxia for a short-term period without the requirement to assisted ventilation and finally was discharged with the good general condition after 2 days.

After hospital discharge, limb jerking movements were noticed during sleep by his parents which were diagnosed with neonatal sleep myoclonus by their family physician. They were stopped within a few days without treatment. In addition, he was admitted again due to exaggerated neonatal jaundice with indirect bilirubin of 17 mg/dL at the end of the first week of life which was treated by phototherapy. Given his developmental milestones, he achieved neck holding at age 2 months, rolling over at age 4–5 months, and starting to crawl at age 6 months.

Thereafter, the patient’s motor development stopped and motor regression commenced. His parents recognized that he lost his ability to crawl and using his legs especially the left foot, gradually. He was not able to sit even with support at 8 months old. As a result, firstly, his parents visited a pediatric orthopedist, and then, they were referred to a child neurologist for further investigation. Moreover, his pelvic X-ray was normal and he had no contracture, joint deformity, and obvious scoliosis. On neurologist examination at 9 months of age, lower limb spasticity, hand fisting, increased deep tendon reflexes (DTR 3+) in lower limbs, and bilateral upward plantar reflex were detected. Ophthalmic examination included all directions’ gaze, fix and follow, reaction to light, and strabismus was normal. His weight and head circumference were 8 kg (*Z*-score −1SD) and 45 cm (*Z*-score 0SD), respectively. All biochemical and basic metabolic tests including serum creatine phosphokinase (CPK), thyroid, liver, and kidney function tests; uric acid level; plasma amino acid chromatography (HPLC); and alpha-1, 4 glucosidase enzyme activity level which was measured by dried blood spot (DBS) test were within normal limit. Occupational therapy was started and a follow-up visit was scheduled at 1 year old.

On review at age of 12 months, his motor ability had declined, significantly. He was not able to crawl or roll over anymore. Furthermore, his lower limbs had become more spastic. He had acceptable social interactions and no significant feeding or swallowing problems, but his speech and cognition skills progression were arrested according to his age. His weight, height, and head circumference were 8.5 kg (*Z*-score −1SD), 76 cm (*Z*-score 0SD), and 46 cm (*Z*-score 0SD), respectively. A brain and spinal MRI were done which were reported normal by a neuroradiologist (not shown).

Additional metabolic studies consisting of serum ammonia and lactate, metabolic screen (MS/MS), acylcarnitine, and urine organic acid profiles were all normal. Abdominal and pelvic ultrasonography revealed an ectopic right kidney in addition to horseshoe kidneys. Cardiologic and ophthalmologic consultations indicated no significant findings. Finally, whole-exome sequencing was done according to motor neurologic regression, speech, and cognitive delay; parents’ consanguinity; normal metabolic test results; and imaging study.

The second brain MRI which was done at 18 months old indicated delayed myelination of subcortical white matter in addition to abnormal signals of posterior periventricular white matter (Fig. 1 (3A, C white arrows)). The T1-weighted sequence at the level of basal ganglia indicated no significant finding (Fig. 1 (3B)). Sagittal T1-weighted image showed mild thinning of the corpus callosum and upper vermis (Fig. 1 (3D)). Furthermore, electromyography and nerve conduction velocity studies (EMG-NCV) revealed generalized sensory polyneuropathy.

### Molecular findings

Data analysis of the called variants in the three unrelated families revealed the genetic etiology of affected individuals. Prioritization of variants led to the identification of three distinct homozygous variants in the *ACER3* gene (NM_018367.7) which they were absent in public variant databases. Sanger sequencing confirmed the segregation of variants within the family members.

The c.53T>C variant found in patient P1 is residing at the first exon of *ACER3* and 18^th^ amino acid of the encoded protein. The c.292T>C variant (in patient P2) is affecting the exon 4 of this gene and results to a substitution in 98^th^ residue of ACER3 protein. Both missense variants were similar regarding to their pathogenicity, which was predicted by in silico pathogenicity prediction tools (Table 2). Examining the level of conservation at the protein level showed that these two variants possess high conservation from humans to yeast. MUPro was also used to analyze the protein stability based on protein Delta G upon the mutation revealing that missense variants lead to diminished protein stability.

**Table 2:** *ACER3* variants and evaluation of its pathogenicity using online prediction tools

Study	Patient	Variant	Gene/genomic location	Allele frequencies	Effect
Current study	A1	NM_018367.7:c.53 T>C Leu18Pro	Chr11(GRCh37):g.76572073T>C	gnomAD: NR	FATHMM-MKL: damaging
				1000 Genome: NR	Mutation Taster: disease causing
				ESP: NR	MutPred: pathogenic
				Iranome: NR	SIFT: damaging
	A2	NM_018367.7:c.292T>C p.Tyr98His	Chr11(GRCh37):g.76687357T>C	gnomAD: NR	FATHMM-MKL: damaging
				1000 Genome: NR	Mutation Taster: disease causing
				ESP: NR	MutPred: pathogenic
				Iranome: NR	SIFT: damaging
	A3	NM_018367.7:c.566G>A p.Trp189*	Chr11(GRCh37):g.76726128G>A	gnomAD: NR	FATHMM-MKL: damaging
				1000 Genome: NR	Mutation Taster: disease causing
				ESP: NR	MutPred: N/A
					SIFT: N/A
Previous studies	P1	NM_018367.7:c.98A>G p.Glu33Gly	Chr11(GRCh37):g. 76572118A>G	gnomAD: NR	FATHMM-MKL: damaging
				1000 Genome: NR	Mutation Taster: disease causing
				ESP: NR	MutPred: pathogenic
				Iranome: NR	SIFT: damaging
	P2	NM_018367.7:c.233G>A p.Trp78*	Chr11(GRCh37):g.76670041G>A	gnomAD: NR	FATHMM-MKL: damaging
				1000 Genome: NR	Mutation Taster: disease causing
				ESP: NR	MutPred: N/A
				Iranome: NR	SIFT: N/A

Unlike the other two missense variants, the third variant discovered in the P3 family was a nonsense variant creating a stop codon resulting in depletion of downstream residues of ACER3 protein.

**Fig. 2:**
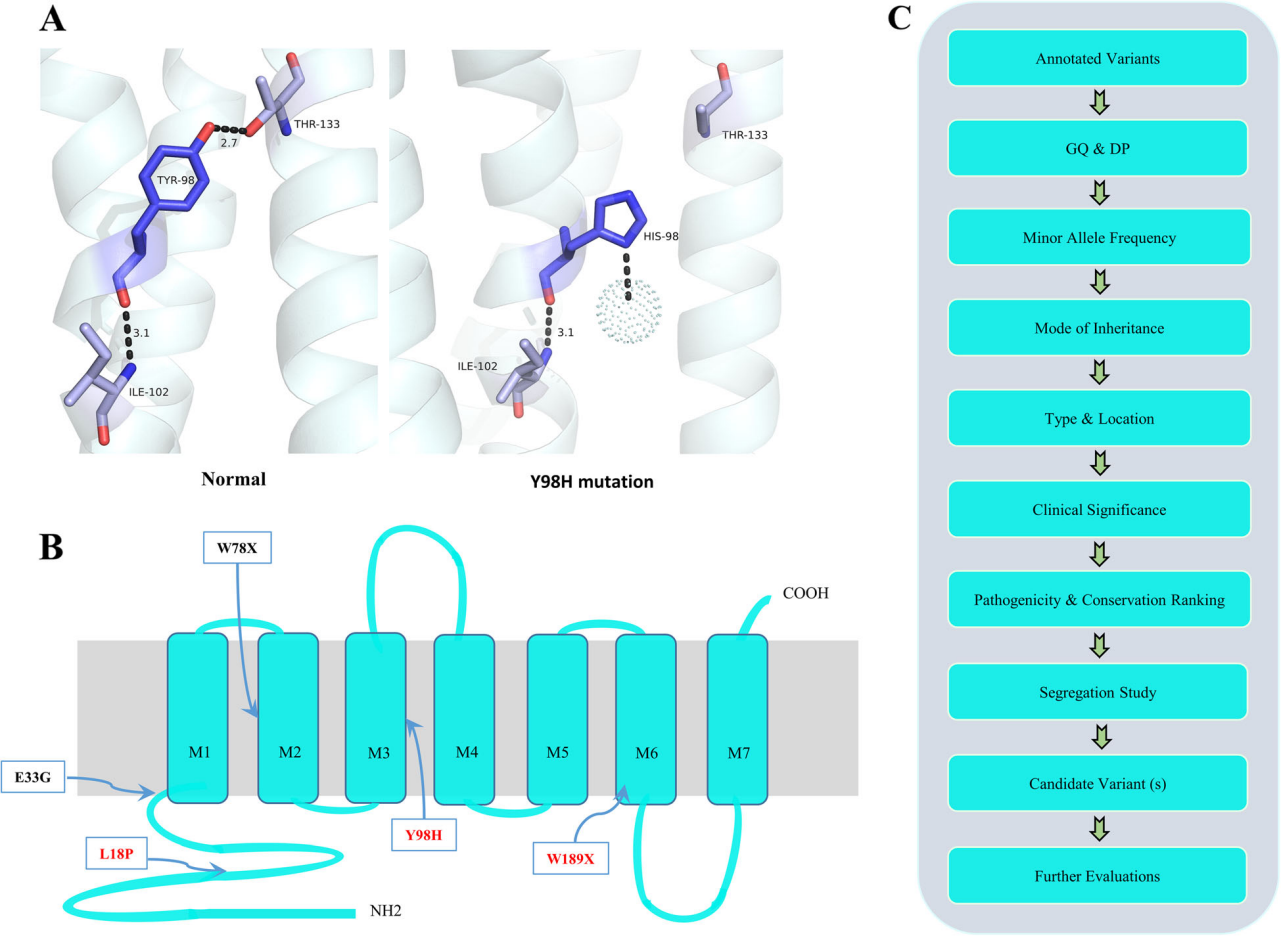
Variant filtering strategy, interactions, and illustration of ACER3 mutations. **A** Precise prioritization scheme was used to identify the disease-causing variant in each understudied family. **B** Studying the interaction of tyrosine residue in normal and mutated state revealed that one putative interaction with Thr133 residue is being lost upon the mutation. **C** The schematic representation of ACER3 protein which is consisted of seven transmembrane domains and several extracellular loops. Two previously reported variants (black) and three novel variants identified in the current study (red) are demonstrated

The available protein structure of ACER3 (6G7O) was utilized to study the interaction state of ACER3 in the normal and mutated form. The visualizations indicated that the interaction of Leu18 with the residues in its vicinity had no alteration after substitution to proline. In contrast, the other variant seemed to lost a strong hydrogen bond to Thr133 (2.7 **Å**) and made a bond with a neighboring water molecule (Fig. 2A).

## Discussion

It has just been recently that homozygous mutations of the *ACER3* gene were found as a culprit for a new form of progressive leukoencephalopathy and established a framework for the clinical window of patients harboring variants in this gene [10, 11]. Having said that, the accumulation of more clinical and genotyping data related to this gene not only could provide a better understanding of the associated phenotypes in the clinical section but helps to provide insights into the genotype-phenotype correlation of *ACER3.*

Here, we report three non-related Iranian families harboring mutations in *ACER3* which were discovered by means of WES causing a progressive neurologic disorder in association with leukoencephalopathy. So far, only two variants have been reported in the *ACER3* gene (Fig. 2B). This study reports the clinical data of the additional disease-causing variants and expands the clinical spectrum of patients carrying these variants.

By studying the crystal structure of the encoded protein of ACER3, it has been shown that the first pathogenic variant discovered in this gene, E33G, leads to protein disability through Ca^2+^ destabilization [12]. It is worth mentioning that E33, W20, D19, N24, E22, and C21 are responsible to ensure the coordination of Ca^2+^. Using the same structure, we investigated protein structure in the mutant state of the identified variants. The L18P as the variant discovered in patient P1 seems to have no effects on hydrogen bonds upon the mutation (interactions are not shown). Interestingly, it is demonstrated that cytoplasmic L18 amino acid is in a hydrophobic interaction network with W20, F80, and W189 residues which are thought to facilitate the functional effect of Ca^2+^ on the catalytic activity of the enzyme by creating a hook-shaped pocket [12]. Thus, this change might interfere with the whole process of ACER3 activation and in turn leading to protein dysfunction. Furthermore, the study of protein structure revealed that the substitution of tyrosine to threonine in 98^th^ residue (located at third transmembrane domain) of ACER protein results in the loss of one putative interaction with T133, which ultimately may disturb the protein stability. Hence, these hypotheses could serve as the explanation for the in silico predicted protein disability and the observed phenotype in our understudy patients but additional functional studies are required to confirm it. Furthermore, the third identified variant is a stop codon mutation (W189*) which eliminates downstream residues within a crucial metal-binding site of ACER3. H217 and H221 which are in interaction with zinc ion and W219 amino acid position which is vital for attachment of the protein to the sodium are among the most important residues in the omitted region.

In this study, all three patients demonstrated developmental delay and neurologic regression started at 5 to 11 months of age mainly including truncal hypotonia, appendicular spasticity, dystonia, infrequent non-febrile seizures, and feeding problem. It was noted that the mean age of onset of clinical symptoms in our three patients was lower than the other reported patients. The other novel finding in terms of the neurological examination was the presence of hyperreflexia, while in other patients decreased deep tendon reflexes were reported. It is worth noting that the occurrence of focal motor type seizures which started at age of 27 months in P1 was another novel clinical finding. A sensory polyneuropathy without motor nerve involvement was detected in P2; however, it was reported in Kilic et al. study as well. Therefore, it is worthy to evaluate the role of ACER3 in peripheral nervous system development. A high arched palate was also a novel minor non-neurologic symptom in P1. Ophthalmic examination was normal in our patients compared with a pale optic disc which was reported in the two last reports [10].

Regarding the clinical course of the disease, all patients including ours experienced apparently normal growth and development followed by symptoms such as stagnation and neurologic regression with relative sparing of cognition landmarks, at least at disease onset. Indeed, considering the initial symptoms of our patients gives a reasonable conclusion in terms of relative sparing of cognitive abilities during the first year of life, but motor and speech milestones seem to be more affected. Besides that, aggravation of neurological decline following vaccination at the age of 18 months in P2 and aspiration pneumonia in P1 was noticeable. Except for the patient of Edvardson et al. study, all patients exhibited acquired late-infantile-onset microcephaly in a similar way.

With respect to imaging findings, faint abnormal signals of periventricular and deep white matter mainly in the parietooccipital area, compensative ventriculomegaly, and progressive supra and infratentorial atrophy and thinning of the corpus callosum were among the cardinal reported findings. Given these imaging findings, it seems that ACER3-related leukoencephalopathy could not be considered as a true leukodystrophy or at least proving this requires more research. This is because the imaging findings of this disorder are more similar to primary neuronal disorders such as neuroid ceroid lipofuscinosis (NCL) rather than real leukodystrophies [13].

## Conclusion

We reported three additional cases of *ACER3-*related leukoencephalopathy with a younger mean age of onset. We added increased DTRs, seizure, and neurologic regression as new clinical findings and delayed myelination under 2 years old and progressive cerebral and cerebellar vermis atrophy as novel imaging findings to the disease spectrum. The presence of peripheral sensory neuropathy and high blood lactate in some cases need to be checked in more patients in future. The clinical follow-ups and serial imaging in our patients indicated that ACER3-related leukoencephalopathy is more compatible with a primary neuronal disorder rather than a leukodystrophy.

## Material and methods

### Ethical statement

The understudy families sought medical counseling at Myelin Disorders Clinic, Children’s Medical Center hospital, and then referred to the Medical Genetics Department of DeNA laboratory for genetic investigation. Written informed consent was obtained from the patient’s parents or guardians, and the ethics review board at Children’s Medical Center hospital, Tehran, Iran, provided approval for this study protocol.

### Molecular analysis

Genomic DNA (gDNA) was extracted from the blood of each individual using the Roche extraction kit (Product No. 11814770001). After quality assessment of extracted gDNA using a nanodrop, fragmentation into the 200–300-bp pieces was done and adapters were ligated to the obtained fragments. Thence, utilizing Agilent’s Sure Select Human All exon V6 kit (Agilent, Santa Clara, CA, USA), the exonic regions and flanking exon-intron boundary regions of the genome of the index patient were captured. High-throughput sequencing was performed for each captured library on Illumina’s HiSeq4000 instrument in accordance with the manufacturer’s protocols (Illumina Inc, USA). Analysis of Exome data has been described before in detail  [14]. In summary, raw reads in FASTQ format were initially aligned onto the hg19 reference genome using BWA 0.7.17 after ensuring the elimination of low-quality reads. Afterward, the aligned reads were processed and variants (SNPs and Indels) were called implementing the Genome Analysis Toolkit 4.1. Annotations were incorporated into the called variants using ANNOVAR [15] (http://annovar.openbioinformatics.org/en/latest/). Prioritization of variants is illustrated in Fig. 2C. Briefly, intragenic, UTR regions, intronic, and synonymous and common variants with allele frequency above 1% (according to 1000 Genomes Project, dbSNPv152, Exome Sequencing Project (ESP), Exome Aggregation Consortium (ExAC) database) were filtered out from the further analysis. Next, pathogenicity evaluator tools (Mutation Taster [16], SIFT [17], Polyphen-2 [18], MutPred2 [19]) were used to rank the remained variants. ClinVar (https://www.ncbi.nlm.nih.gov/clinvar) and HGMD (http://www.hgmd.cf.ac.uk) were used for the assessment of genotype-phenotype correlation of variants and their relation to patient’s medical history and clinical information. Sanger sequencing was carried out by using ABI 3500xL DNA Analyzer (Applied Biosystems, Foster City, CA, USA) to confirm/exclude the candidate variants. The bidirectional primers listed in Supplementary Table 1 are utilized for PCR amplification of the variant region. Finally, sequencing traces were analyzed using the SnapGene v.5.0.5 software. The variants were classified according to the American College of Medical Genetics (ACMG; http://wintervar.wglab.org/) guidelines [20].

### Conservation study, protein structure stability, and interactions

The Human ACER3 protein structure (PDB ID: 6G7O) was employed to study the polar contacts of the natural and mutated residue by PyMOL software (The PyMOL Molecular Graphics System, Version 2.3.2, Schrödinger, LLC). MUPro was utilized to calculate the protein structure stability upon the mutation by implementing a support vector machine [21]. The evolutionary conservation profile of ACER protein was determined using the UCSC database and Consurf [22].

## Supplementary Information

**Additional file 1: Supplementary Table S1.** Sequences of the primers used to confirm the identified variant by Sanger sequencing.

## Data Availability

Human variant and pertinent phenotypes have been reported to ClinVar (accession numbers: SCV001448219, SCV001448218, SCV001479340.1). The whole-exome sequencing dataset used and analyzed in the current study is available from the corresponding author upon request.
