# Supplementary material for: ACER3-related leukoencephalopathy: expanding the clinical and imaging findings spectrum due to novel variants
Source: Hum Genomics. 2021 Jul 19;15:45. doi: 10.1186/s40246-021-00345-0 (PMC8287746; doi:10.1186/s40246-021-00345-0)
Supplement: Supplementary file 1 — Additional file 1: Supplementary Table S1. Sequences of the primers used to confirm the identified variant by Sanger sequencing. [file 40246_2021_345_MOESM1_ESM.docx]

**Supplementary Table S1.** Sequences of the primers used to confirm the identified variant by Sanger sequencing

| Gene | Variant | Primers |
| --- | --- | --- |
| *ACER3* | c.53T>C | F: 5′- GTCAGGGCAGTGTCAGTAACG -3′ |
|  |  | R: 5′- TCCCTCTACACACTGGACTCC -3′ |
|  | c.292T>C | F: 5′- TGCTGCTCCATGATATTCAAGC -3′ |
|  |  | R: 5′- TTCTGGTAAGCGTCTTGTTAAAC -3′ |
|  | c.566G>A | F: 5′- TGGCTTCAATTTCTGATCGTGAC -3′ |
|  |  | R: 5′- ACAGATCAGGCATGTTTCAAAGG -3′ |
